# Traumatic Events and Vaccination Decisions: A Systematic Review

**DOI:** 10.3390/vaccines10060911

**Published:** 2022-06-08

**Authors:** Maria Christou-Ergos, Kerrie E. Wiley, Julie Leask, Gilla K. Shapiro

**Affiliations:** 1 Susan Wakil School of Nursing and Midwifery, Faculty of Medicine and Health, University of Sydney, Camperdown 2006, Australia; julie.leask@sydney.edu.au; 2 Sydney School of Public Health, Faculty of Medicine and Health, University of Sydney, Camperdown 2006, Australia; kerrie.wiley@sydney.edu.au; 3 Sydney Institute for Infectious Diseases, Westmead Hospital, Westmead 2145, Australia; 4 Department of Supportive Care, Princess Margaret Cancer Centre, Toronto, ON M5G 2C1, Canada; gilla.shapiro@uhnresearch.ca

**Keywords:** psychological trauma, vaccination, review

## Abstract

Despite the apparent relationship between past experiences and subsequent vaccination decisions, the role of traumatic events has been overlooked when understanding vaccination intention and behaviour. We conducted a systematic review to synthesize what is known about the relationship between traumatic events and subsequent vaccination decisions. MEDLINE, PsycINFO and CINHAL electronic databases were searched, and 1551 articles were screened for eligibility. Of the 52 articles included in full-text assessment, five met the eligibility criteria. Findings suggest that the experience of trauma is associated with individual vaccination decisions. Social and practical factors related to both trauma and vaccination may mediate this relationship. As this is a relatively new field of inquiry, future research may help to clarify the nuances of the relationship. This review finds that the experience of psychological trauma is associated with vaccination intention and behaviour and points to the potential importance of a trauma-informed approach to vaccination interventions during the current global effort to achieve high COVID-19 vaccine coverage.

## 1. Introduction

Past experiences are an influential factor in the decision to vaccinate [1,2]. More generally, an individual’s thoughts and behaviour can be affected by their experience of a traumatic event [3]. Traumatic events are experiences that place an individual or someone close to them at risk of serious harm or death [3]. An intensely distressing event that does not pose the risk of serious harm or death is a stressful event. While the prevalence of specific experiences of trauma varies globally, many individuals will experience a traumatic event at some point in their lives [4,5]. Thus, exploring the role of trauma in influencing vaccination decisions is of potential importance when understanding vaccination intention and behaviour. The decision to vaccinate and the consequences of a traumatic experience are each related to cognitive appraisals, social factors and control beliefs, suggestive of a relationship between the two.

Psychological distress following exposure to a traumatic event is variable [3,6,7]. Posttraumatic stress disorder (PTSD) is a psychiatric disorder that may occur in people who have experienced or witnessed a traumatic event, so long as they also experience (for over a month): (a) intrusive symptoms associated with the traumatic event (e.g., flashbacks, nightmares, recurrent memories); (b) persistent avoidance; (c) alterations in mood; and d) increased arousal/reactivity (e.g., outbursts of anger or irritability, lack of concentration, sleep disturbances) [3]. The experience of traumatic events may affect threat appraisal and outcome predictions, which are also utilised when making vaccination decisions. Appraisals that underlie vaccination decisions are based on outcome predictions and perceived threats. Namely, this involves the perceived susceptibility to a vaccine preventable disease (VPD), the perceived severity of the VPD [8] and the anticipated regret of contracting a VPD following vaccine refusal [9]. Similarly, traumatic events have consequences for decision-making via alterations in threat appraisal mechanisms [10] by which maladaptive interpretations and memories of trauma induce a sense of threat in everyday situations [10]. 

Social factors and control beliefs underpin both the decision to vaccinate and the consequences of trauma. Several studies show a relationship between social norms and vaccination intentions [11,12,13,14]. Norms within social networks are evidenced through the geographical clustering of vaccine objectors [15,16] and congruence of vaccine attitudes within families [17,18]. Similarly, social factors such as low social support may heighten the consequences of trauma [19,20] and, inversely, relationships may be affected by traumatic events [21]. The interaction between subjective norms and perceived behavioural control shows a strong association with vaccination intention. Since trauma is often associated with feelings of loss of control and/or helplessness, which may impact volitional control [22], this suggests yet another way in which vaccination decisions may relate to the experience of psychological trauma. 

Current global vaccination efforts demand an understanding of the drivers of vaccination as well as reasons for under-vaccination in the face of the COVID-19 pandemic. Despite suggestion of an association between psychological trauma and vaccination decisions, the details of this relationship have been overlooked by vaccination interventions. The objective of this systematic review is to synthesize the literature regarding the relationship between traumatic events and vaccination decisions. This may inform whether tailored approaches to address vaccine hesitancy are warranted for trauma-affected individuals.

## 2. Materials and Methods

### 2.1. Search Strategies

A review was conducted in accordance with PRISMA criteria [23]. The online databases MEDLINE, PsycINFO and CINHAL were searched. As this review aimed to understand vaccination decisions following trauma, exemplars of the latter listed in the Diagnostic and Statistical Manual of Mental Disorders (DSM-5) [3] served as a reference point for included terms. The terms ‘fear’ and ‘anxiety’ were added to broaden the search to include studies that investigated related stressors. Terms pertaining to vaccination were few, in order to capture the unique decision to vaccinate as opposed to other related concepts. However, the more general search term “needle *” was included to broaden the search to include effects that may impact vaccination decisions in cases where it was not a primary outcome variable of the research. The Medical Subject Headings (MeSH) terms used to search for articles in Medline were: ‘Psychological Trauma’ or ‘Sexual Trauma’ or ‘Domestic Violence’ or ‘Child Abuse’ or ‘Child Abuse, Sexual’ or ‘Elder Abuse’ or ‘Spouse Abuse’ or ‘Gun Violence’ or ‘Intimate Partner Violence’ or ‘Physical Abuse’ or ‘Rape’ or ‘Terrorism’ or ‘Anxiety’ or ‘Fear’ or ‘Combat Disorders’. Text word terms were: ‘Psychological Trauma’ or ‘Sexual Trauma’ or ‘Violence’ or ‘war’ or ‘abuse *’ or ‘assault’ or ‘rape’ or ‘terroris *’or ‘accident *’ or ‘disaster *’ or ‘anxiety’ or ‘fear *’ and ‘vaccin *’ or ‘immuniz *’ or ‘immunis *’ or ‘needle *’. 

### 2.2. Inclusion and Exclusion Criteria

Inclusion criteria were for peer reviewed studies involving human subjects, published in English between 1980 and 2021 which reported on vaccination of individuals who had experienced trauma. This timeframe was selected since 1980 marked the introduction of post-traumatic stress disorder as a diagnosis in the third iteration of the DSM [24], and thus was the year that trauma was officially recognised for its potential to clinically affect the individual, as it did not appear in the International Classification of Diseases (ICD) until 1992 [25]. The review excluded studies for which data collection commenced before 1980, in which subjects were not human, and those that were not written in English. Studies were included if they explored both (i) a traumatic event and (ii) vaccination decisions. The search strategy excluded studies concerning general psychological outcomes (e.g., depression or anxiety) without reference to trauma and studies investigating concepts related to other needle procedures (e.g., phlebotomy, injected medication or other injection paraphernalia). In the few cases where there was ambiguity regarding a study meeting these criteria, two additional reviewers (J.L. and K.E.W.) assessed the study for deliberation until consensus was reached.

### 2.3. Screening

**Figure 1:**
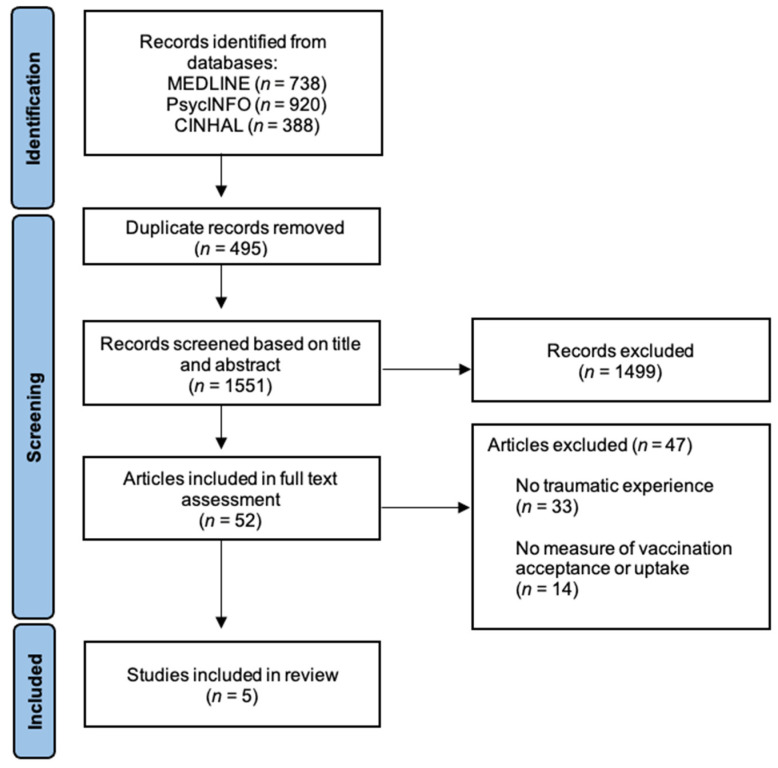
Flowchart of selection process of articles included in the review.

Following the removal of duplicates, studies were screened for inclusion based on review of the title and abstract. Remaining studies underwent a full-text assessment against inclusion and exclusion criteria with references within relevant articles also screened in the same manner; first by title and abstract, and then by full text assessment. Following screening (Figure 1), five studies were included in the review. 

## 3. Results

### 3.1. Study Characteristics

Five studies were included in this review. Table 1 summarises the study type, location by country, and sample, as well as the traumatic events referenced in relation to vaccination decisions, population affected by the events, vaccine being studied, vaccination decision agent (i.e., the person responsible for the vaccination decision), and the key findings relating to trauma and vaccination.

### 3.2. Findings

The review identified few studies that comment on a traumatic experience in reference to vaccination decisions. Two studies found that vaccine acceptance was associated with perceived likelihood of VPD infection amplified by a traumatic event. A cross-sectional survey of 461 females [26] reported an increase in acceptance of the HPV associated with an increased experience of violence (91.1% vs. 80%, *p* < 0.021) including emotional (91.9% vs. 83.7%, *p* < 0.027) or physical violence (90.6% vs. 84.8%, *p* < 0.05). Another qualitative study [27] found that a heightened fear of cholera during a humanitarian crisis was related to increased acceptance of a cholera vaccine. Trust was of particular importance in the latter study, whereby distrust in institutions was associated with hesitancy, while inversely, increased trust was associated with acceptance. However, unlike all other studies in this review which measure vaccine uptake, these two studies measure vaccination intention. As intentions and behaviours have been found to differ within populations [31], the comparison of these findings with others in this review is tentative.

Three studies examined a traumatic event and vaccine uptake [28,29,30]. A cross-sectional survey of 124,385 women [28] found that maternal experience of physical and/or sexual interpersonal violence was associated with decreased likelihood of full immunization among their children (RR = 0.90; 95% CI = 0.83–0.98) although it did not explore the reason for this relationship. Similarly, vaccine refusal was evident in a study of pediatric survivors of sexual assault [29] in which 48% of vaccine eligible patients did not receive the HPV vaccine during the intervention. This study identifies limited social support via the absence of a consenting and guiding caregiver as a key barrier to vaccine uptake. A population level cross-sectional survey examined vaccination uptake following a ferry disaster with far-reaching, pervasive effects on mental health and social disruption [30]. Residents of a comparison city received more vaccination than residents in the city affected by the disaster (AOR = 1.10; 95% CI = 1.04–1.17; *p* = 0.002). While there was no difference in the vaccination rates between depressed individuals in both cities (AOR = 0.90; 95% CI = 0.75–1.09; *p* = 0.281), non-depressed individuals residing in the same locality as disaster victims received fewer vaccinations following the disaster (AOR = 1.12; 95% CI = 1.05–1.20; *p* < 0.001) compared to non-depressed individuals in the comparison city.

## 4. Discussion

Overall, there is limited research investigating the specific relationship between the experience of a traumatic event and vaccination. The aim of this review is to consolidate what is known. The studies included in this review are relatively recent, with the oldest published in 2012. This suggests that the exploration of the subject is new and that more research is needed to make definitive conclusions. Nevertheless, the findings of this review suggest that the experience of traumatic events is associated with vaccination decisions, and that decisions may be influenced by social and practical factors that are related to both the traumatic event and vaccination experience.

While three of the studies reviewed [28,29,30] found that the experience of a traumatic event was associated with decreased vaccination, two studies [26,27] found that interpersonal violence and a humanitarian crisis were associated with increased vaccination against HPV and cholera, respectively. This suggests that risk appraisals may depend on the type of trauma and vaccine in question, along with other potential factors that require further investigation. Interestingly, the two studies that found that a traumatic experience was associated with increased vaccine acceptance were the only two studies that measured vaccination intention rather than uptake. Thus, vaccination intention and behaviour may differ following a traumatic experience, and while individuals may be very motivated to receive a vaccine, practical barriers may affect vaccine uptake.

Studies included in this review were conducted in India (*n* = 1), South Korea (*n* = 1), South Sudan (*n* = 1), and the United States of America (*n* = 2), and thus were diverse in cultural scope. As such, the influence of contextual factors may be relevant when drawing conclusions from this review. Vaccination decisions are influenced by social and cultural factors [32], as are the consequences of traumatic events that can be shaped by cultural [33] and gender norms [34]. The mediating effect of these variables in relation to trauma and vaccination may be an appropriate avenue for future research.

It is difficult to compare vaccination decisions in this review, as decision agents and vaccine target groups differ among the samples. While most studies of traumatic experiences in this review report on personal vaccination decisions, two studies focused on parents’ vaccination decisions for their children. Generally, surrogate decision-making may alter risk appraisals so that they differ from those underlying decisions made for oneself [35,36]. The experience of trauma notwithstanding, the decision to vaccinate a child may be made by more than one parent or caregiver, thus adding a social element to the decision-making process. The social role of decision agents in the context of a traumatic event requires further evaluation in future research.

At an individual level, the mechanism underlying the effect of traumatic experiences on vaccination decisions has not been explicitly considered. Although only explicitly mentioned by one qualitative study in this review [27], vaccination research indicates that confidence, underpinned by trust, is a moderate correlate of vaccine acceptance [37]. Since the experience of trauma may affect an individual’s capacity for trust [22], this is a potentially important consideration for vaccination decisions following trauma. While most studies considered traumatic events experienced by individuals directly, one study investigated the vaccination behaviour of individuals following death or harm caused to others by a disaster [31]. While this might be due to practical challenges imposed on the community, this might also be due to vicarious trauma acquisition which can also affect an individual’s decision making [3]. Indeed, experiencing first-person narratives of victims are found to influence behaviour [38]. The impact of vicarious trauma on vaccination decisions may be important when seeking to understand vaccine hesitancy. This may be especially important in the face of anti-vaccination rhetoric that uses personal anecdotes of traumatic vaccination experiences as evidence of alleged vaccine harms [39,40,41].

The small number of heterogenous studies conducted, limits the generalizability of our conclusions, as does our limited scope of inquiry. Our search terms pertaining to trauma were not exhaustive and focused on exemplars of discrete traumatic events listed by the DSM-V which are thus most likely to have clinical implications. This was done to provide the most pointed findings. Accordingly, studies relating to trauma that are not defined as such, but may nonetheless have subjective psychological implications for individuals, were excluded. We note that constructs were not always well defined by studies that were screened and that conceptual clarity is an issue in the literature. There is a large body of literature concerning medically distressing events. Medical experiences that qualify as traumatic events involve sudden, catastrophic events (e.g., waking during surgery, anaphylactic shock) [3]. There were many studies concerning negative vaccination procedures that were excluded. While a painful or negative vaccination experience may be a stressor, it may be considered traumatic if it causes the individual serious physical harm and is accompanied by other factors (see above). Broadening the search to encompass stressful events may add to our conclusions; however, this is reliant on the reporting of the impact of trauma on individual subjects.

The included studies did not measure symptoms associated with PTSD in individuals who experienced trauma. People who have experienced a broad range of traumatic events follow different trajectories in their subsequent functioning in that they are either (i) resilient, (ii) gradually recovering after an initial period of distress, (iii) worse as time progresses, or (iv) chronically distressed [42,43,44,45]. Moreover, traumatic events that are pervasive or experienced in childhood can have greater effects on daily functioning [46]. Thus, understanding the symptoms currently experienced by individuals and the timing of the traumatic event relevant to vaccination may help to draw conclusions about how the consequences of traumatic events may be associated with vaccination decisions. Moreover, there is a need for studies to clearly operationalize traumatic events, experienced symptoms, and vaccination outcomes for a more complete understanding of this relationship. Future research should pay close consideration to the person making the vaccination decision, vaccine target group, and trauma-affected group of interest to better understand the mechanism at an individual level. Finally, studies in this review reveal associations between traumatic experiences and vaccination decisions but do not attempt to understand underlying mechanisms.

Our work adds to the growing recognition of the relationship between traumatic experiences and medical encounters [47]. Various trauma-informed approaches to communication within primary health care have facilitated an individualized approach [48,49,50], and qualitative vaccination studies suggest that vaccine-hesitant individuals value individualized management and communication [51,52]. This review highlights the potential importance of considering trauma history prior to vaccination. While various psychometric tools exist to screen for trauma exposure, further research may help decide the best screening method to employ in conjunction with immunization delivery services. Moreover, future research should investigate the efficacy of interventions that use a trauma-informed approach on vaccination intention and uptake. While there are many models of trauma informed healthcare, there are no prescribed actions for providing a trauma-informed service. Rather, these encompass a shift in in the way providers think about trauma and interact with patients [53]. Generally, trauma-informed approaches recognize that trauma is a widespread experience that can affect all levels of the medical context, and respond to this by applying trauma knowledge into practice and endeavoring to prevent further trauma [54]. Future research could consider the most cost-and time-effective actions to implement alongside vaccination procedures under such an approach.

## 5. Conclusions

This review makes apparent the overlooked and potentially important role of psychological trauma in shaping vaccination intention and behaviour. This is a relatively new field of inquiry and, as such, more research is needed to explore this relationship and understand its mechanism. This review suggests that vaccination interventions may benefit from understanding the unique experiences and perspectives of trauma-affected individuals. Research that focuses on the efficacy of a trauma-informed approach to vaccination delivery services may be helpful for guiding efforts to address vaccine hesitancy.

## Figures and Tables

**Table 1:** Studies relating to traumatic experiences and vaccination.

Study	Country	Study Type	Sample	Traumatic Experience	Affected Population	Type of Vaccination	Vaccination Decision Agent	Key Findings
Champion, 2017 [26]	United States of America	Cross-sectional Survey	Mexican- American Females aged 14–18 (n = 461)	Emotional and physical interpersonal violence	Child	Human Papilloma Virus (HPV)	Child	Greater vaccine acceptance associated with increased experience of violence.
Peprah et al., 2016 [27]	South Sudan	Qualitative	Internally displaced adults (n = 49)	Humanitarian disaster	Adult self	Cholera	Adult self	Heightened fears of disease and political danger contributed to increased vaccine acceptance.
Sabarwal et al., 2012 [28]	India	Cross-sectional Survey	Women aged 15–49 years (n = 124,385)	Intimate partner violence	Parent	Child vaccination (0–2 years)	Parent	Decreased likelihood of full immunization among children of mothers who experienced violence.
Goodman & Goodpasture, 2020 [29]	United States of America	Quality Improvement Intervention	Children aged > nine (n = 111)	Child sexual abuse	Child	HPV	Parent	Almost half of the children who experienced abuse did not receive a vaccine during the intervention.
Kang, 2020 [30]	South Korea	Cross-sectional Survey	Adults aged ≥19 (n = 11,026)	Ferry disaster	Adult self	Influenza	Adult self	Residents in the city affected by the disaster received less vaccination than comparison city residents.

## Data Availability

Not applicable.
